# 4-Phenylbutyric Acid Attenuates Pancreatic Beta-Cell Injury in Rats with Experimental Severe Acute Pancreatitis

**DOI:** 10.1155/2016/4592346

**Published:** 2016-08-30

**Authors:** Yu-pu Hong, Wen-yi Guo, Wei-xing Wang, Liang Zhao, Ming-wei Xiang, Fang-chao Mei, Ablikim Abliz, Peng Hu, Wen-hong Deng, Jia Yu

**Affiliations:** ^1^Department of General Surgery, Renmin Hospital of Wuhan University, 238 Jiefang Road, Wuhan, Hubei Province 430060, China; ^2^Key Laboratory of Hubei Province for Digestive System Disease, 9 Zhangzhidong Road, Wuhan, Hubei Province 430060, China; ^3^Central Laboratory, Renmin Hospital of Wuhan University, 9 Zhangzhidong Road, Wuhan, Hubei Province 430060, China

## Abstract

Endoplasmic reticulum (ER) stress is a particular process with an imbalance of homeostasis, which plays an important role in pancreatitis, but little is known about how ER stress is implicated in severe acute pancreatitis (SAP) induced pancreatic beta-cell injury. To investigate the effect of 4-phenylbutyric acid (4-PBA) on the beta-cell injury following SAP and the underlying mechanism, twenty-four Sprague-Dawley rats were randomly divided into sham-operation (SO) group, SAP model group, and 4-PBA treatment group. SAP model was induced by infusion of 5% sodium taurocholate into the biliopancreatic duct. 4-PBA or normal saline was injected intraperitoneally for 3 days in respective group before successful modeling. Results showed that 4-PBA attenuated the following: (1) pancreas and islet pathological injuries, (2) serum TNF-*α* and IL-1*β*, (3) serum insulin and glucose, (4) beta-cell ultrastructural changes, (5) ER stress markers (BiP, ORP150, and CHOP), Caspase-3, and insulin expression in islet. These results suggested that 4-PBA mitigates pancreatic beta-cell injury and endocrine disorder in SAP, presumably because of its role in inhibiting excessive endoplasmic reticulum stress. This may serve as a new therapeutic target for reducing pancreatic beta-cell injury and endocrine disorder in SAP upon 4-PBA treatment.

## 1. Introduction

Severe acute pancreatitis (SAP) is an inflammatory disorder characterized by an intricate cascade of events originating from digestive enzyme activation in pancreas acinar cells [1–3]. Pancreas, a gland which has both exocrine and endocrine functions with a plenty of secretory cells, depends on high endoplasmic reticulum (ER) volume and functionality owing to massive protein synthesis and processing [4]. Pancreatic islets scatter among the acinar cells and consist mainly of beta cells which secrete insulin, the unique hormone lowering blood glucose level. Pancreatic beta cells are susceptible to disturbance in ER homeostasis [5].

ER stress is provoked by the accumulation of partially folded or misfolded proteins in the ER lumen [6] and activates unfolded protein response (UPR). Increasing evidence has demonstrated that ER stress and UPR are relevant in numerous pathophysiological disorders including sepsis [7], ulcerative colitis [8], rheumatoid arthritis [9], systemic lupus erythematosus [10], neurodegenerative diseases [11], diabetes mellitus [12], and viral infection [13]. In addition, ER stress may play a role in acute pancreatitis and associated beta-cell injury [14, 15]. The ER chaperone heavy chain binding protein (BiP), also known as glucose regulated protein 78 (GRP78), is anchored to ER transmembrane proteins. When ER homeostasis is disrupted, BiP binds to unfolded or misfolded proteins to assist folding and is transcriptionally upregulated through the UPR to alleviate homeostasis [16]. The 150 kDa oxygen regulated protein (ORP150) belongs to the heat-shock family 70 and is upregulated by ER stress as a chaperone protein [17]. The CCAAT/enhancer-binding protein homologous protein (CHOP) is a specific marker of ER stress and UPR activation as a proapoptotic transcription factor [18]. When the injury is excessive and invincible, CHOP is overexpressed to trigger cell death. And prolonged ER stress conditions can activate the apoptosis effector Caspase-3 [15].

4-Phenylbutyric acid (4-PBA) is a short-chain fatty acid with three main pharmacological effects as an ammonia scavenger [19], a weak histone deacetylase inhibitor (HDACi) [20], and an ER stress inhibitor [21]. 4-PBA has been approved for clinical use in patients suffering urea cycle enzyme deficiencies and hyperammonemia, where it acts as an ammonia scavenger [19, 22, 23]. Nonetheless, it has been widely studied as a small chemical chaperone to modulate restoration of ER homeostasis and multiple concerning pathological conditions [12, 24, 25].

However, whether 4-PBA is effective in ameliorating pancreatic islet beta-cell damage in SAP* in vivo* has not yet been elucidated. The aim of the present study was to evaluate the effect of 4-PBA on beta-cell injury following SAP as well as the underlying mechanisms.

## 2. Materials and Methods

### 2.1. Animals and Regents

Adult male SPF Sprague-Dawley rats, with weight from 200 g to 250 g, were provided by Hunan SJA Laboratory Animal Co. (Changsha, Hunan, China). All animals were housed under standardized environment with free access to standard laboratory rodent chow and sterile water. The study was approved by the Committee on Ethics of Animal Experiments of Wuhan University and performed in compliance with the Guide for the Care and Use of Laboratory Animals from the National Institutes of Health. 4-PBA and sodium taurocholate (STC) were obtained from Sigma-Aldrich Co. (St. Louis, MO, USA). Isoflurane was from Abbott Laboratories (Shanghai, China). EliVision super/horseradish peroxidase (HRP) kits were purchased from Maixin Biotech Co. (Fuzhou, China). Rat tumor necrosis factor-*α* (TNF-*α*) and interleukin-1*β* (IL-1*β*) enzyme-linked immunosorbent assay (ELISA) kits were purchased from eBioscience Inc. (Vienna, Austria). Rat insulin ELISA kit was from Elabscience Biotechnology Co. (Wuhan, China). BiP, ORP150, CHOP, and Caspase-3 antibodies were purchased from Abcam (Cambridge, United Kingdom); insulin antibody was from Cell Signaling Technology Inc. (Danvers, MA, USA).

### 2.2. Experimental Model and Groups

Rats were deprived of food for 12 h prior to the experiment but given water* ad libitum.* After the body weight of all animals was measured, anesthesia to all rats was induced with 4% isoflurane in 2 L/min oxygen in a sealed container and was maintained with 2% isoflurane in 2 L/min oxygen during surgery. The SAP rat model was induced by a standardized retrograde infusion of 5% sodium taurocholate (STC) saline solution at a dose of 1 mL/kg body weight into the biliopancreatic duct at a velocity of 0.1 mL/min after laparotomy. The abdominal incision was closed and 20 mL/kg of saline was injected into the back subcutaneously to compensate for the fluid loss.

The rats were randomly divided into three groups. (1) 4-PBA treatment group (4-PBA group, *n* = 8): 4-PBA solution was prepared as described earlier and minor steps were revised as follows [26]: equimolar amounts of 4-PBA and sodium hydroxide were titrated to pH 7.4 and dissolved in physiological saline. 4-PBA was administered* via *intraperitoneal injection at a dose of 50 mg/kg per day for 3 days from 10:00 a.m. to 1:00 p.m. After last injection SAP was induced. (2) Severe acute pancreatitis model group (SAP group, *n* = 8): rats received a volume-matched physiological saline substituted for 4-PBA before SAP. (3) Sham-operation group (SO group, *n* = 8): in this group, rats received a sham surgery where the pancreas and duodenum were flipped a number of times instead of STC infusion.

### 2.3. Blood and Tissue Procurement

Rats from each group were sacrificed at 12 h after treatment. Blood samples were collected by inferior vena cava puncture and centrifuged at 4,000 ×g for 10 min, and the serum was stored at −20°C in individual aliquots. Following sacrifice, the head of the pancreas tissue was immediately excised and fixed in 4% phosphate-buffered formaldehyde for hematoxylin-eosin (H&E) staining and immunohistochemical detection.

### 2.4. Histopathological Examination

For histopathological analysis, paraffin-embedded pancreatic tissues were sequentially sliced at 4 *μ*m thick and treated with H&E staining. The morphologic evaluation of the pancreatic tissues was performed under optical microscope (Olympus Optical Ltd., Tokyo, Japan) by two experienced pathologists who were blinded to the experimental groups. Pancreatic histological assessment was scored for the severity of pancreatitis based on edema, inflammation and hemorrhage, and acinar necrosis according to the standard scale described by Schmidt et al. [27].

### 2.5. Serum Assay

Serum amylase (AMY), lipase (LIPA), and glucose levels were measured using standard techniques with a fully automatic chemistry analyzer (ADVIA 2400 Clinical Chemistry System; Siemens Healthcare Diagnostics Inc., New York, USA). Serum insulin, TNF-*α*, and IL-1*β* levels were measured by the use of commercially available ELISA kits according to the manufacturer's instructions. The absorbance was determined by an automated microplate ELISA reader (PERKINELMER Ltd., Waltham, Mass, USA) and concentrations were computed by the standard curve run on each assay plate. All samples were measured in duplicate.

### 2.6. Immunohistochemical Analysis

Immunohistochemical analyses of BiP, ORP150, CHOP, Caspase-3, and insulin in paraffin-embedded pancreas sections (4 *μ*m) were performed using a standard streptavidin-peroxidase (SP) method and were modified as follows [28]. Following xylene deparaffinisation and hydration using a graded series of ethanol solutions: the slides were boiled for 4 min at 121°C in a pressure cooker containing 10 mM citrate buffer (pH 6.0) for the epitope retrieval. Subsequently the slides were cooled to room temperature and rinsed in phosphate-buffered saline (PBS). Endogenous peroxidase activity was blocked by 3% hydrogen peroxide for 15 min. Incubating the section in 5% normal goat serum in PBS for minimizing nonspecific staining: the primary antibody was a rabbit polyclonal antibody to BiP (1 : 300) and Caspase-3 (1 : 50) and monoclonal antibody to ORP 150 (1 : 150), CHOP (1 : 100), and insulin (1 : 200). Sections were incubated with the primary antibody overnight at 4°C in a humidity box and then incubated with a second antibody. The staining results were visualized using the 3,5-diaminobenzidine. Then the slides were counterstained with hematoxylin, dehydrated through an ethanol series, and mounted with coverslips. The negative control experiments were performed in which PBS was substituted for the primary antibody. All sections were examined and photographed using light microscope (×400) under blind conditions.

Immunohistochemical staining was analyzed by Image Pro-Plus 6.0 system (Media Cybernetics, Inc., Rockville, MD, USA) for quantitative analysis. Briefly, the islet was selected as the area of interest (AOI). The measurement parameters were the area sum and integrated optical density (IOD) of the AOI. The mean optical density (MOD) of BiP, ORP150, CHOP, and Caspase-3 and insulin expression in islets of different groups were analyzed and compared.

### 2.7. Transmission Electron Microscopy (TEM)

Pancreatic beta-cell ultrastructure was examined by a transmission electron microscope. A small portion (about 1 mm^3^) of fresh tissue was excised from the pancreas tail and fixed in 2.5% glutaraldehyde (0.1 mol/L phosphate buffer, pH 7.4) over night at 4°C, followed by postfixation with 1% osmium tetroxide in the same buffer for 1 h at 4°C. The tissues were dehydrated in a graded series of ethanol and acetone. Ultrathin sections were cut on Leica EMUC7 ultramicrotome, stained with lead citrate and uranyl acetate, and changes of the beta cells were examined by a HT7700 transmission electron microscope (Hitachi, Japan).

### 2.8. Statistical Analysis

Data were presented as means ± standard deviation. The data were analyzed with SPSS 20.0 statistical software for Windows (SPSS Inc., Chicago, USA). Differences between groups were compared by one-way analysis of variance (ANOVA). The correlations of insulin with TNF-*α*, IL-1*β*, and glucose were analyzed using Spearman correlation test. A value of *P* < 0.05 was considered statistically significant.

## 3. Results

### 3.1. Serum AMY and LIPA Levels

**Figure 1:**
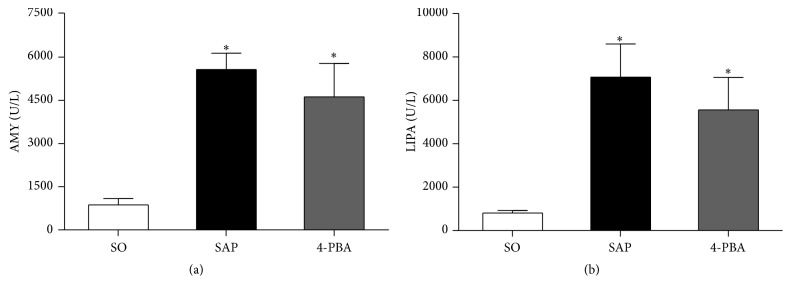
Serum levels of AMY, LIPA in all groups of rats. Each value represents the mean ± standard deviation. ^*∗*^ *P* < 0.05 versus SO group.

As shown in Figure 1, compared with SO group, serum AMY and LIPA levels were significantly increased at 12 h in SAP group (*P* < 0.05). Pretreatment with 4-PBA reduced the serum levels of AMY and LIPA compared with those in SAP group, but the difference was not significant (*P* > 0.05).

### 3.2. Histopathological Analysis of Pancreas and Islet

**Figure 2:**
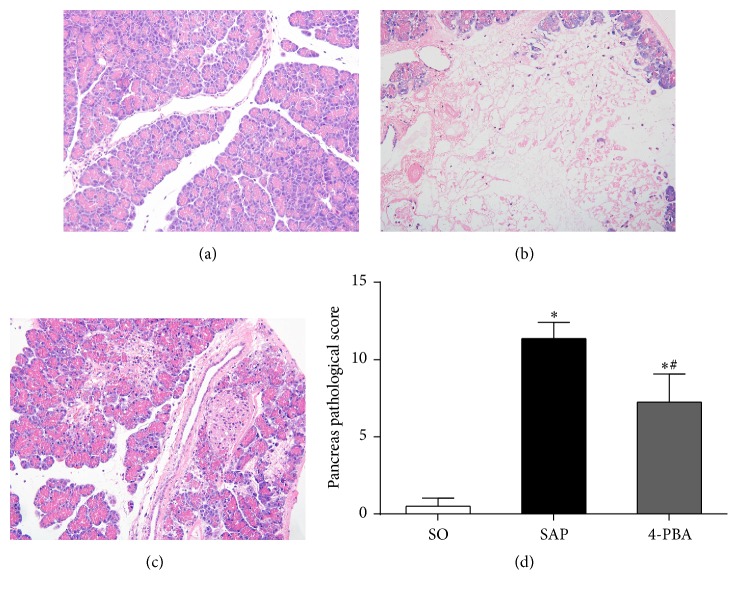
Morphologic changes in pancreatic tissue at 12 hours and pancreatic histopathological scores in all groups of rats. Representative hematoxylin and eosin-stained sections were examined by light microscopy (original magnification, ×200). (a) Sham-operation (SO) group; (b) severe acute pancreatitis model (SAP) group; (c) 4-PBA treatment (4-PBA) group. (d) Comparison of the total pathological scores of the pancreas in all groups. Each value represents the mean ± standard deviation. ^*∗*^ *P* < 0.05 versus SO group; ^#^ *P* < 0.05 versus SAP group.

Representative changes of pancreatic tissue are shown in Figure 2. Little morphological evidence of pancreatic injury except mild interstitial edema was observed in SO group. In SAP group, massive areas of acinar necrosis, inflammatory cell infiltration, and hemorrhage were observed. Compared with SAP group, the pancreatic histopathological score significantly decreased in the 4-PBA group (*P* < 0.05).

**Figure 3:**
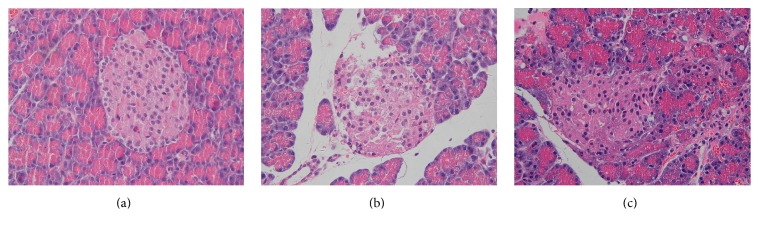
Morphologic changes in pancreatic islet at 12 hours in all groups of rats. Representative hematoxylin and eosin-stained sections were examined by light microscopy (original magnification, ×400). (a) SO group; (b) SAP group; (c) 4-PBA group.

Representative changes of pancreatic islets are shown in Figure 3. Many pancreatic islet cells clustered to mass with well-defined boundary from the exocrine pancreas in SO group. These cells were equally distributed, plump rounded, with clear margins and oval nuclei placed in the middle. In SAP group, islet had an irregular-shaped appearance, with some vacuolization and a significant reduction in number and size of islet cells. Variably sized nuclei with some pyknotic nuclei were detected as well. An unclear boundary between islet and the exocrine pancreas was observed in 4-PBA group; however, when pretreated with 4-PBA, the vacuolar degeneration, karyopyknosis, and number of islet cells were improved as compared to SAP group.

### 3.3. Serum Insulin, TNF-*α*, IL-1*β*, and Glucose Levels

**Figure 4:**
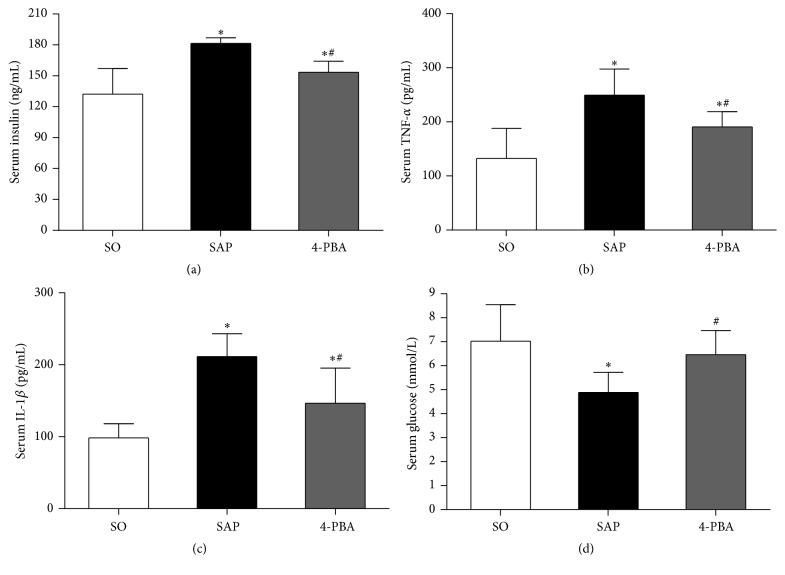
Effects of 4-phenylbutyric acid on insulin, inflammatory cytokine, and glucose production in serum. (a) Insulin; (b) TNF-*α*; (c) IL-1*β*; (d) glucose. Each value represents the mean ± standard deviation. ^*∗*^ *P* < 0.05 versus SO group; ^#^ *P* < 0.05 versus SAP group.

As illustrated in Figure 4, in SAP group, serum concentrations of insulin, TNF-*α*, and IL-1*β* were markedly increased compared with those in SO group (*P* < 0.05). By contrast, serum insulin, TNF-*α*, and IL-1*β* levels were significantly reduced by the administration of 4-PBA compared with SAP group (*P* < 0.05). Compared with SO group, serum level of glucose was decreased significantly in SAP group (*P* < 0.05). Pretreatment with 4-PBA elevated blood glucose level compared with SAP group (*P* < 0.05).

**Figure 5:**
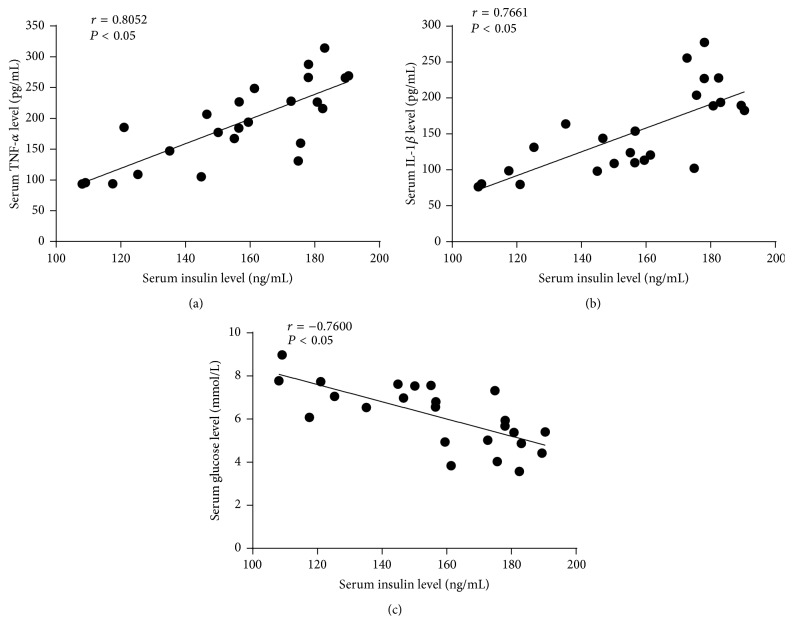
The correlations of serum insulin with TNF-*α*, IL-1*β*, and glucose were analyzed using Spearman correlation test. (a) Spearman correlation between insulin and TNF-*α*; (b) Spearman correlation between insulin and IL-1*β*; (c) Spearman correlation between insulin and glucose.

All data of serum insulin, TNF-*α*, IL-1*β*, and glucose levels were used to calculate correlation coefficient. Spearman correlation analysis revealed that serum levels of insulin were positively correlated with TNF-*α* (*r* = 0.8052, *P* < 0.05) and IL-1*β* (*r* = 0.7661, *P* < 0.05) and showed significantly negative correlation between serum insulin levels and serum glucose levels (*r* = −0.7600, *P* < 0.05) (Figure 5).

### 3.4. Immunohistochemical Analysis

**Figure 6:**
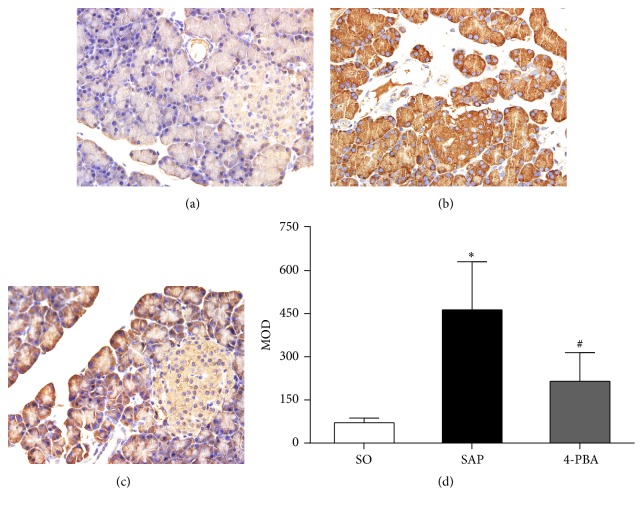
Immunohistochemical expression of BiP in pancreatic islets (original magnification, ×400). (a) SO group; (b) SAP group; (c) 4-PBA group. (d) Comparison of mean optical density (MOD) of BiP in all groups. Each value represents the mean ± standard deviation. ^*∗*^ *P* < 0.05 versus SO group; ^#^ *P* < 0.05 versus SAP group.

**Figure 7:**
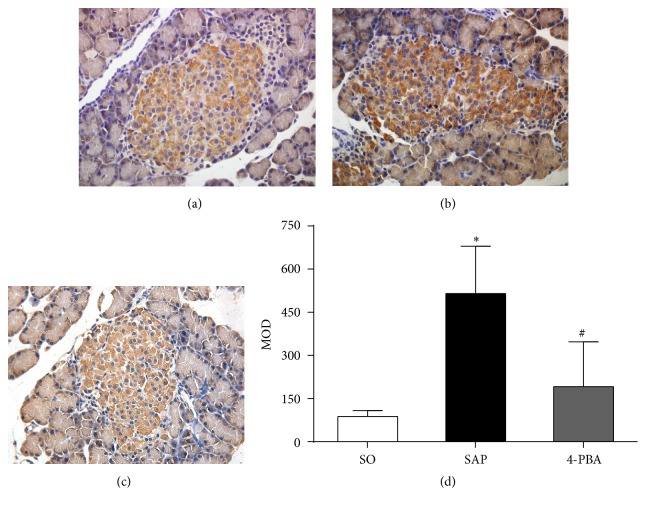
Immunohistochemical expression of ORP150 in pancreatic islets (original magnification, ×400). (a) SO group; (b) SAP group; (c) 4-PBA group. (d) Comparison of MOD of ORP150 in all groups. Each value represents the mean ± standard deviation. ^*∗*^ *P* < 0.05 versus SO group; ^#^ *P* < 0.05 versus SAP group.

**Figure 8:**
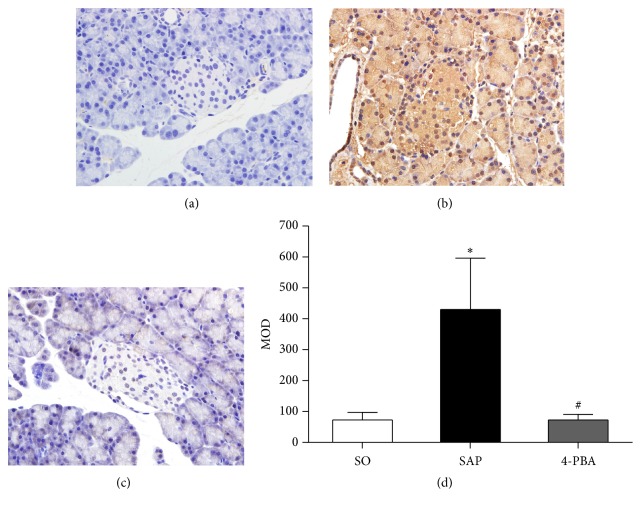
Immunohistochemical expression of CHOP in pancreatic islets (original magnification, ×400). (a) SO group; (b) SAP group; (c) 4-PBA group. (d) Comparison of MOD of CHOP in all groups. Each value represents the mean ± standard deviation. ^*∗*^ *P* < 0.05 versus SO group; ^#^ *P* < 0.05 versus SAP group.

**Figure 9:**
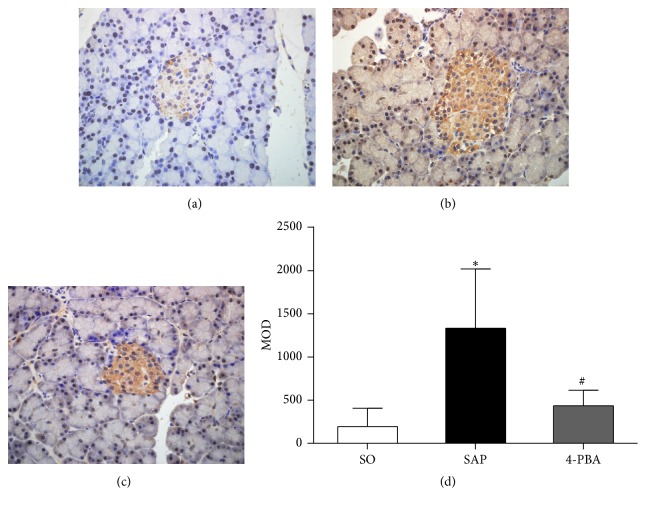
Immunohistochemical expression of Caspase-3 in pancreatic islets (original magnification, ×400). (a) SO group; (b) SAP group; (c) 4-PBA group. (d) Comparison of MOD of Caspase-3 in all groups. Each value represents the mean ± standard deviation. ^*∗*^ *P* < 0.05 versus SO group; ^#^ *P* < 0.05 versus SAP group.

**Figure 10:**
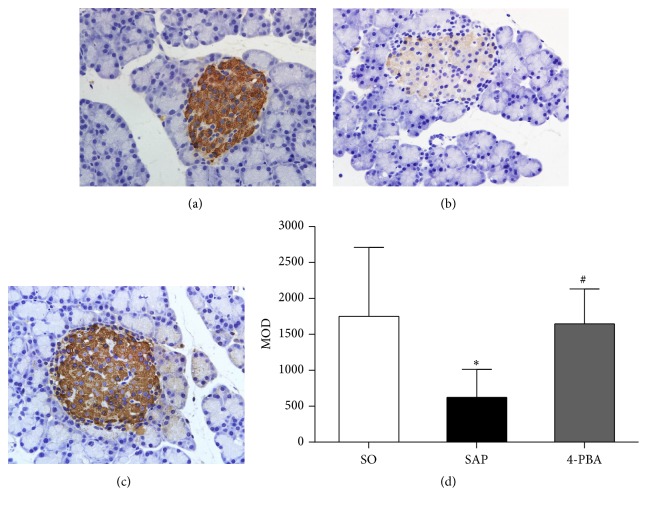
Immunohistochemical expression of insulin in pancreatic islets (original magnification, ×400). (a) SO group; (b) SAP group; (c) 4-PBA group. (d) Comparison of MOD of insulin in all groups. Each value represents the mean ± standard deviation. ^*∗*^ *P* < 0.05 versus SO group; ^#^ *P* < 0.05 versus SAP group.

Immunohistochemical assay was conducted to evaluate the expressions of BiP, ORP150, CHOP, Caspase-3, and insulin in pancreatic islets. In SO group, the weak expressions of BiP in pancreatic islets were observed; in contrast, BiP was overexpressed in the islets in SAP group; positive immunostaining for BiP was significantly reduced in rats pretreated with 4-PBA (*P* < 0.05; Figure 6). The expressions of ORP150 were weak in the islets of sham-operation rats, and there was intense immunoreactivity in the islets in SAP group. In 4-PBA group, however, a marked reduction in ORP150 staining in the islets was observed (*P* < 0.05; Figure 7). In SAP group, there was a significant rise in the positive staining for CHOP in the islets compared with SO group, while a markedly weaker immunoreactivity of CHOP was detected in 4-PBA group (*P* < 0.05; Figure 8). Caspase-3 was expressed weakly in the islets in SO group and overexpressed in SAP group; however, significantly lower expressions of Caspase-3 were observed in 4-PBA group compared with SAP group (*P* < 0.05; Figure 9). An abundance of insulin granules was well distributed in the middle of islets in SO group. After injection of STC, insulin expressions in islets at 12 h were weaker than SO group, and pretreatment of 4-PBA increased the expressions of insulin in islets compared with those in SAP group (*P* < 0.05; Figure 10).

### 3.5. Ultrastructural Examination of Pancreatic Beta Cells

**Figure 11:**
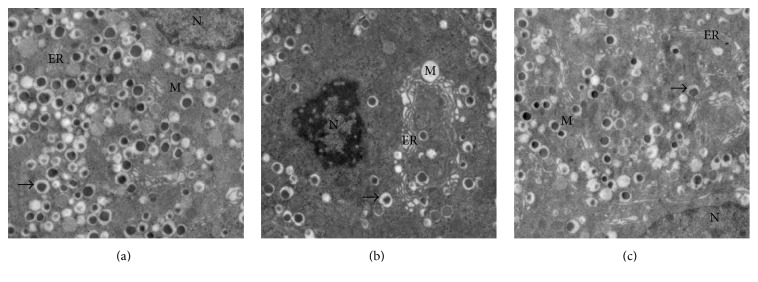
Electron microscopic analysis of pancreatic beta cells in different groups. (a) Ultrastructure of pancreatic beta cell showed that the nuclei, endoplasmic reticulums, mitochondria, and secretory granules were not abnormal in SO group (original magnification, ×3,000). (b) The changes of pancreatic beta cells in SAP group show condensed chromatin and loss of secretory granules, concomitant with severely dilated endoplasmic reticulums and swollen mitochondria (original magnification, ×3,000). (c) Mild dilatation of the endoplasmic reticulums, decrease of mitochondria damage, and increase of secretory granules were noted in 4-PBA group (original magnification, ×3,000).* Note*. Nucleus (N), mitochondria (M), endoplasmic reticulum (ER), and secretory granules (black arrow).

The ultrastructural changes of beta cells from representative islet are shown in Figure 11. Pancreatic beta cells in SO rats showed normal morphology of nuclei, endoplasmic reticulum, and mitochondria. The cells contained abundant secretory granules, which were composed of an electron dense core, an external membrane, and a space between them (Figure 11(a)). In SAP rats, condensation and margination of nuclear chromatin markedly dilated ER cisternae and swollen mitochondria were present in beta cells. The number of secretory granules was significantly decreased compared to SO group (Figure 11(b)). In 4-PBA treated rats, TEM examination has revealed that beta cells had euchromatic nuclei, slightly dilated cisternae of ER, increased mitochondrial matrix density, and secretory granules (Figure 11(c)). These suggested that ultrastructural damage of pancreatic islet beta cells was ameliorated by 4-PBA pretreatment.

## 4. Discussion

The development of SAP is a complex process that initiates in the pancreatic acinar cells and is characterized by inflammation response and cell death, usually accompanying disorder of endocrine function as a result of pancreatic islet injury [29, 30]. ER stress and its responses participate in the development of acute pancreatitis [31]. Under the circumstance of unresolved and severe ER stress, persistent activation of the UPR may destroy the pancreatic acinar cell and beta-cell homeostasis that constantly end in cell death owing to accumulation of misfolded or unfolded proteins and subsequently result in release of digestive enzymes and inflammatory cytokines, as well as insulin [32, 33].

4-PBA, a Food and Drug Administration-approved drug, because of its chemical chaperone property by improving ER stress, has been used as a therapeutic agent of diabetes associated beta-cell failure [21, 34] and some inflammatory diseases [35, 36] on cellular as well as organismal level. In addition, this is the first* in vivo* study, to our knowledge, detailing an islet especially beta cells protective effect of 4-PBA in SAP. In the present study, the increasing levels of serum AMY, LIPA, TNF-*α*, IL-1*β*, and insulin and decreasing levels of serum glucose after SAP were observed. These findings were consistent with our previous research, which demonstrated that a plenty of insulin was released from pancreatic beta cells into the blood as the body was under stress reaction during SAP [37]. The prophylactic administration of 4-PBA reduced the serum levels of proinflammatory mediators and insulin and increased the serum levels of glucose following SAP. This is also supported by the decreased injury of pancreatic acini and islets in light microscopy. Otherwise, a negative correlation between serum insulin levels and serum glucose levels was detected, and serum levels of insulin were positively correlated with TNF-*α* and IL-1*β*. The results suggested that pancreatic endocrine disorder was associated with the development of SAP. On the other hand, beta cells with sparse and light insulin staining were shown in SAP rats, while 4-PBA added the insulin staining significantly in the survival islets. At the same time, decreased number of secretory granules in beta cells by TEM examination and increased insulin levels in serum following SAP were detected, while 4-PBA raised the number of secretory granules in beta cells and reduced insulin levels in serum. These findings indicated that 4-PBA could mitigate the severity of SAP, and moreover 4-PBA reduced destruction of islets caused by SAP and preserved residual pancreatic islet beta-cell function by constraining excessive release of stored insulin and consequently relieved the SAP-related pancreatic endocrine disorder.

As cellular homeostasis is destroyed, a series of signals were immediately activated to cope with stress by promoting the synthesis of chaperone proteins. Previous study has demonstrated that activation of BiP improved the capacity of protein folding and processing during ER stress [38]. Moreover, ORP150 has been reported as an ER-resident molecular chaperone which facilitated insulin release [39]. Excessive ER stress leads to overexpression of CHOP that is hardly expressed under physiological conditions [40]. An obvious increase in the expressions of BiP, ORP150, CHOP, and Caspase-3 in islets was observed following SAP at 12 h, indicating the development of ER stress. However, application of 4-PBA made a great reduction of their expressions. Furthermore, condensed chromatin concomitant with markedly dilated ER cisternae and increase of mitochondria damage in pancreatic beta cells were observed using TEM in SAP rats, and 4-PBA eased the ultrastructural injury induced by SAP. These results suggested that ER stress responses, indicated by GRP78 and CHOP expression, in the pancreatic beta cells were greatly increased after SAP was induced. 4-PBA could inhibit ER stress by suppressing expression of not only BiP, ORP150, and CHOP, but also Caspase-3, which is a key enzyme in the apoptosis process. Further, 4-PBA might protect against SAP-related pancreatic endocrine disorder by stabilizing the cellular homeostasis and preventing islet apoptotic cell death by means of modulating ER stress.

## 5. Conclusions

The present study demonstrates that ER stress is involved in pancreatic beta-cell injury and pancreatic endocrine dysfunction after attack of severe acute pancreatitis. A chemical chaperone, 4-PBA, ameliorates the beta-cell injury and dysfunction by protecting the islet structure, controlling synthesized insulin, and inhibiting beta-cell apoptosis and necrosis mediated by ER stress. The results obtained from this study suggest that the ER stress response can act as a mediator for pancreatic endocrine disorder observed in SAP patients. Although this work found that 4-PBA can exert beneficial effects for SAP in rats by its cytoprotective properties to restrain ER stress-related inflammation and cell death after SAP, the accurate molecular mechanism of 4-PBA modulated ER stress is recommended to investigate in further research. As 4-PBA has been in clinical use, this study provides a theoretical basis that 4-PBA can be a new therapeutic strategy restoring pancreatic endocrine function in connection with severe acute pancreatitis.
