# Corrigendum to “4-Phenylbutyric Acid Attenuates Pancreatic Beta-Cell Injury in Rats with Experimental Severe Acute Pancreatitis”

**DOI:** 10.1155/2018/6307830

**Published:** 2018-01-09

**Authors:** Yu-pu Hong, Wen-yi Guo, Wei-xing Wang, Liang Zhao, Ming-wei Xiang, Fang-chao Mei, Ablikim Abliz, Peng Hu, Wen-hong Deng, Jia Yu

**Affiliations:** ^1^Department of General Surgery, Renmin Hospital of Wuhan University, 238 Jiefang Road, Wuhan, Hubei Province 430060, China; ^2^Key Laboratory of Hubei Province for Digestive System Disease, 9 Zhangzhidong Road, Wuhan, Hubei Province 430060, China; ^3^Central Laboratory, Renmin Hospital of Wuhan University, 9 Zhangzhidong Road, Wuhan, Hubei Province 430060, China

In the article titled “4-Phenylbutyric Acid Attenuates Pancreatic Beta-Cell Injury in Rats with Experimental Severe Acute Pancreatitis” [1], the authors made a mistake in the process of analysis by using the recommended concentrations of reference standard which should be diluted by 20 times to be the actual concentrations.

Therefore, in the “3.3. Serum Insulin, TNF-*α*, IL-1*β*, and Glucose Levels” section, the text reading “Spearman correlation analysis revealed that serum levels of insulin were positively correlated with TNF-*α* (*r* = 0.8052, *P* < 0.05) and IL-1*β* (*r* = 0.7661, *P* < 0.05) and showed significantly negative correlation between serum insulin levels and serum glucose levels (*r* = −0.7600, *P* < 0.05) (Figure 5)” should be corrected as follows.

“Spearman correlation analysis revealed that serum levels of insulin were positively correlated with TNF-*α* (*r* = 0.8070, *P* < 0.05) and IL-1*β* (*r* = 0.8270, *P* < 0.05) and showed significantly negative correlation between serum insulin levels and serum glucose levels (*r* = −0.7191, *P* < 0.05) (Figure 5).”

In addition, the raw data for Figures 4(a) and 5 are included in the Supplementary Materials (available here) and the figures should be corrected as follows.

## Figures and Tables

**Figure 4:**
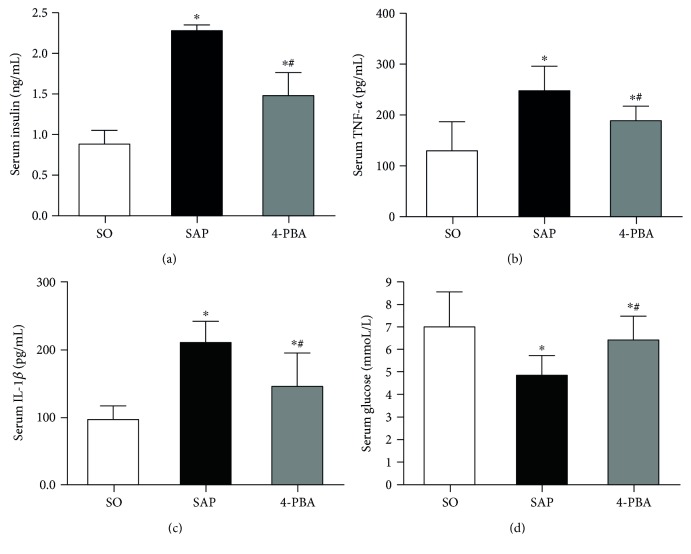
Effects of 4-phenylbutyric acid on insulin, inflammatory cytokine, and glucose production in serum. (a) Insulin; (b) TNF-*α*; (c) IL-1*β*; (d) glucose. Each value represents the mean ± standard deviation. ^∗^ *P* < 0.05 versus SO group; ^#^ *P* < 0.05 versus SAP group.

**Figure 5:**
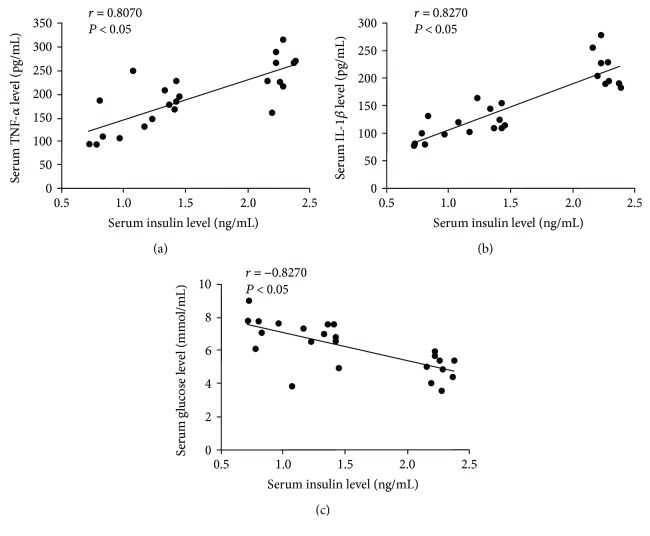
The correlations of serum insulin with TNF-*α*, IL-1*β*, and glucose were analyzed using Spearman correlation test. (a) Spearman correlation between insulin and TNF-*α*; (b) Spearman correlation between insulin and IL-1*β*; (c) Spearman correlation between insulin and glucose.

## Supplementary Materials

Supplementary Materials Table 1: serum levels of insulin, TNF-*α*, IL-1*β*, and glucose in rats.
