# Supplementary material for: Corrigendum to “4-Phenylbutyric Acid Attenuates Pancreatic Beta-Cell Injury in Rats with Experimental Severe Acute Pancreatitis”
Source: Int J Endocrinol. 2018 Jan 9;2018:6307830. doi: 10.1155/2018/6307830 (PMC5818923; doi:10.1155/2018/6307830)
Supplement: Supplementary Materials — Table 1: serum levels of insulin, TNF-α, IL-1β, and glucose in rats. [file 6307830.f1.docx]

| group | insulin (ng/ml) | TNF-α (pg/ml) | IL-1β (pg/ml) | glucose (mmol/L) |
| --- | --- | --- | --- | --- |
| SO group |  |  |  |  |
| rat1 | 0.84 | 108.86 | 131.27 | 7.05 |
| rat2 | 0.81 | 185.22 | 79.53 | 7.74 |
| rat3 | 0.72 | 93.53 | 76.30 | 7.78 |
| rat4 | 1.08 | 248.68 | 120.66 | 3.84 |
| rat5 | 0.97 | 105.28 | 98.01 | 7.62 |
| rat6 | 0.73 | 95.53 | 80.30 | 8.98 |
| rat7 | 0.78 | 93.84 | 98.58 | 6.08 |
| rat8 | 1.17 | 130.77 | 102.25 | 7.32 |
| SAP group |  |  |  |  |
| rat1 | 2.37 | 265.97 | 189.67 | 4.42 |
| rat2 | 2.26 | 226.34 | 189.13 | 5.38 |
| rat3 | 2.23 | 287.58 | 277.29 | 5.94 |
| rat4 | 2.29 | 314.15 | 193.82 | 4.87 |
| rat5 | 2.23 | 266.54 | 227.22 | 5.68 |
| rat6 | 2.38 | 268.97 | 182.67 | 5.4 |
| rat7 | 2.20 | 159.65 | 203.78 | 4.03 |
| rat8 | 2.28 | 216.01 | 227.76 | 3.57 |
| 4-PBA group |  |  |  |  |
| rat1 | 2.16 | 228.07 | 255.52 | 5.02 |
| rat2 | 1.42 | 226.77 | 153.77 | 6.8 |
| rat3 | 1.45 | 193.95 | 113.28 | 4.94 |
| rat4 | 1.42 | 184.06 | 109.99 | 6.56 |
| rat5 | 1.33 | 206.77 | 143.77 | 6.98 |
| rat6 | 1.36 | 177.27 | 108.85 | 7.54 |
| rat7 | 1.41 | 167.27 | 123.85 | 7.56 |
| rat8 | 1.23 | 147.27 | 163.85 | 6.54 |

Table1 Serum levels of insulin, TNF-*α*, IL-1*β*, and glucose in rats
